# Supplementary material for: The interactome of intact mitochondria by cross-linking mass spectrometry provides evidence for coexisting respiratory supercomplexes
Source: Mol Cell Proteomics. 2017 Dec 8;17(2):216–32. doi: 10.1074/mcp.RA117.000470 (PMC5795388; doi:10.1074/mcp.RA117.000470)
Supplement: Supplemental Data [file supp_RA117.000470_133922_0_supp_23978_fzffwf.zip › spectra_annotation/mito_DR_spectra_annotation/226-1-19-1-9-1.pdf]

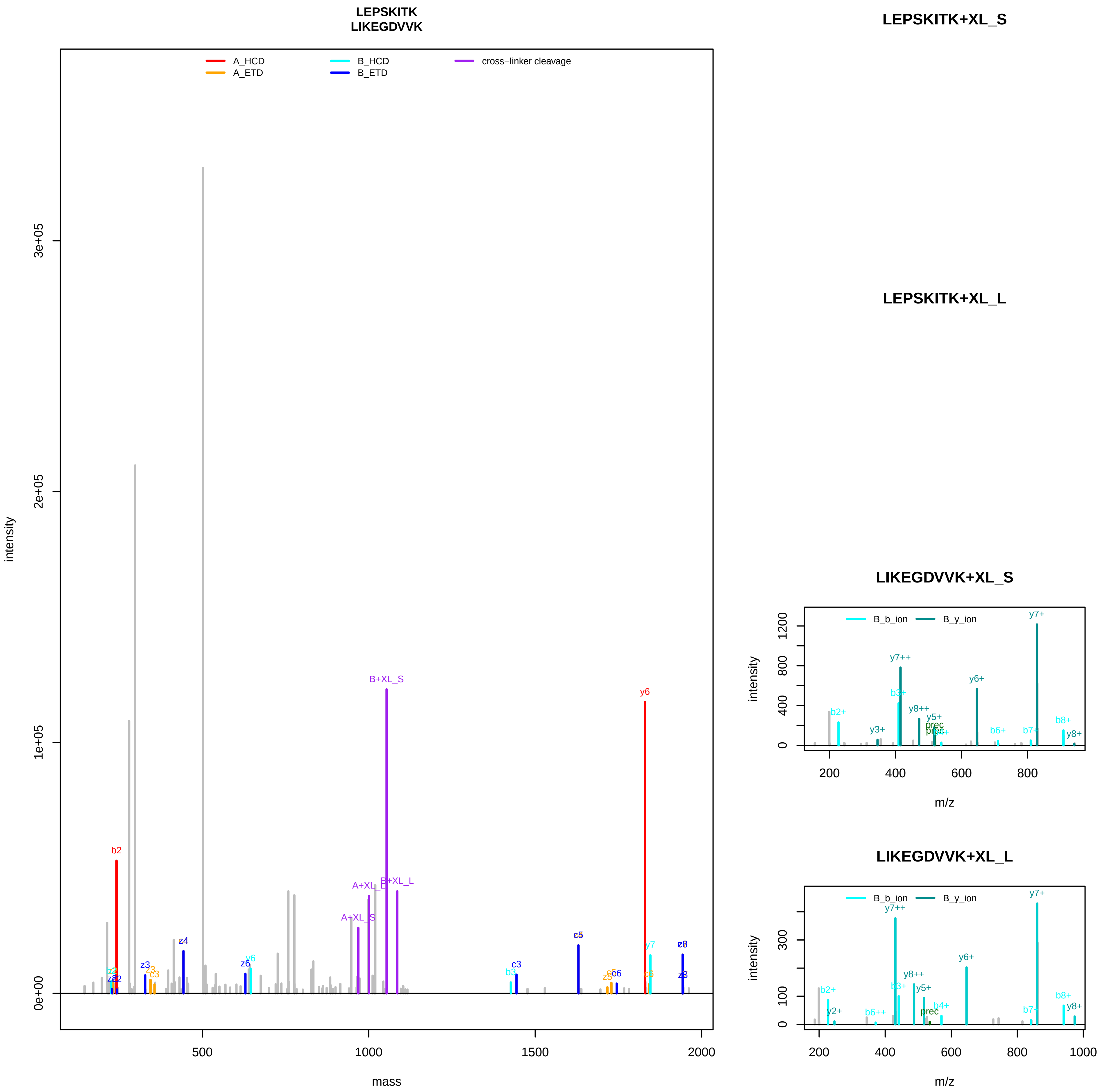
LEPSKITK
LIKEGDVVK
A_HCD
A_ETD
B_HCD
B_ETD
cross−linker cleavage
intensity
mass
0e+00
1e+05
2e+05
3e+05
500
1000
1500
2000
b2
z3
z4
z6
y6
A+XL_S
A+XL_L
B+XL_S
B+XL_L
b3
c3
c5
y6
y7
c6
z8
LEPSKITK+XL_S
LEPSKITK+XL_L
LIKEGDVVK+XL_S
B_b_ion
B_y_ion
y7+
y7++
b3+
y8++
y6+
b2+
y3+
y5+
prec
b6+
b7+
b8+
y8+
m/z
LIKEGDVVK+XL_L
y2+
b6++
b4+
